# Mucosal Adenovirus-Vectored Rv2299c Vaccine Protects Against Tuberculosis by Inducing Trained Immunity in Dendritic Cells and Polyfunctional T Cells

**DOI:** 10.3390/vaccines14010055

**Published:** 2026-01-02

**Authors:** Huiling Wang, Shiqi Xie, Shaoqiong Huang, Xuejiao Huang, Ying Zhang, Juan Wu, Xiao-Yong Fan, Zhidong Hu

**Affiliations:** Shanghai Public Health Clinical Center & Shanghai Institute of Infectious Diseases and Biosecurity, Fudan University, 2901 Cao Lang Road, Jinshan District, Shanghai 201508, China; 23211300002@m.fudan.edu.cn (H.W.); 23211300006@m.fudan.edu.cn (S.X.); 24211300002@m.fudan.edu.cn (S.H.); 25111020024@m.fudan.edu.cn (X.H.); 22111300008@m.fudan.edu.cn (Y.Z.); 22111300001@m.fudan.edu.cn (J.W.)

**Keywords:** tuberculosis, vaccine, trained immunity, dendritic cells, polyfunctional T cells

## Abstract

**Background**: The development of effective tuberculosis (TB) vaccines beyond BCG remains an urgent global health priority, especially for prevention of pulmonary TB in adults. While most current strategies focus on enhancing T-cell immunity, the potential of trained immunity to broadly augment both innate and adaptive responses remains underexplored in TB vaccinology. Given the central role of dendritic cells (DCs) as bridges between innate and adaptive immunity, we hypothesized that inducing trained immunity in DCs could optimize subsequent T-cell responses. Previous studies have identified Rv2299c as a promising adjuvant of other antigens by promoting DC maturation; however, whether it could be used as a standalone protective antigen of TB vaccine remains unclear. **Methods**: We constructed a chimpanzee adenovirus-vectored TB vaccine candidate expressing Rv2299c (rAd-Rv2299c), and evaluated its immunogenicity and protective efficacy in murine models. **Results**: rAd-Rv2299c vaccine effectively induced a trained immunity phenotype in DCs, as evidenced by upregulated MHC-II and CD86 expression and increased pro-inflammatory cytokine (TNF-α, IL-6, IL-1β and IL-12p70) secretion. Moreover, its immunization promoted the generation of antigen-specific polyfunctional T cells, and robustly enhanced both Th1 and Th17-type immune responses. In a murine challenge model, vaccination significantly reduced bacterial loads in the lung and spleen and attenuated pulmonary inflammation, which was associated with robust recall T-cell immune responses. **Conclusions**: rAd-Rv2299c confers anti-TB protection by inducing trained immunity in DCs and promoting polyfunctional T-cell responses, thereby offering valuable experimental evidence and conceptual insights for the development of next-generation TB vaccines.

## 1. Introduction

As the only licensed tuberculosis (TB) vaccine, Bacillus Calmette–Guérin (BCG) effectively prevents severe forms of childhood TB; however, its protective efficacy in adults is limited [1,2]. Considering that studies on anti-TB immunity have firmly established the essential role of T-cell-mediated immunity in controlling *Mycobacterium tuberculosis* (*Mtb*) infection, a central strategy in designing an effective TB vaccine is to elicit robust and durable T-cell-mediated immune responses.

Under this strategy, the screening and identification of *Mtb* antigens are based on T-cell recognition. Classical vaccine antigens such as Ag85A, Ag85B, and ESAT-6 were selected on this basis and have been extensively studied [3,4,5,6]. However, the first novel TB vaccine expressing Ag85A that entered phase IIb clinical trials induced robust antigen-specific CD4 T-cell immunity but failed to demonstrate efficacy against infection [7], highlighting the need for novel approaches to identify protective antigens. Recently, Rv2299c has been explored as a “DC-stimulating adjuvant” to enhance the immune responses induced by other vaccine antigens. Previous studies have shown that fusion proteins incorporating Rv2299c, such as Rv2299c–Ag85A, Rv2299c–ESAT-6 and Rv2299c–Ag85A-ESAT-6, markedly promote DC maturation and enhance Th1 cytokine production, conferring considerable protection against *Mtb* infection in murine models [8,9,10]. These findings suggest that Rv2299c may serve as an adjuvant antigen capable of driving protective T-cell responses through DC optimization. Nevertheless, its potential as a standalone antigen in a vaccine formulation requires systematic evaluation within an effective delivery platform.

Beyond classical adaptive immunity, the emerging concept of “trained immunity” has introduced a paradigm in vaccinology. Trained immunity refers to a long-term functional reprogramming of innate immune cells—such as monocytes, macrophages, and DCs—following exposure to specific stimuli, leading to enhanced nonspecific protection against subsequent infections [11,12]. As pivotal bridges linking innate and adaptive immunity, DCs that acquire a trained phenotype are expected to exhibit enhanced antigen-presenting capacity and pro-inflammatory cytokine secretion, thereby optimizing the activation and functional differentiation of CD4^+^ and CD8^+^ T cells [13,14,15,16]. Thus, vaccines designed to harness DC trained immunity and amplify T-cell responses represent a promising and innovative strategy.

Besides that, recombinant adenoviral vectors have emerged as a preferred vaccine platform due to their high transduction efficiency, ability to mimic natural infection, and potent induction of CD8^+^ and CD4^+^ T cell responses via cross-presentation [17,18]. Human adenovirus type 5 (Ad5)-vectored vaccines, for instance, have been shown to elicit robust T-cell responses against a range of pathogens, including HIV, Zika, Ebola, and *Mtb* [19,20,21,22]. In TB vaccine research, Ad5-based vaccines expressing Ag85A have proven safety and immunogenicity in preclinical and clinical studies, though their protective efficacy requires further optimization. Notably, beyond their established role in adaptive immunity, adenoviral vectors (Ad) have also been shown to induce trained immunity in innate immune cells such as alveolar macrophages [23]. To circumvent the issue of pre-existing immunity to Ad5 in human populations, this study employs a chimpanzee adenovirus vector, which exhibits low seroprevalence in humans and has demonstrated substantial protective potential in TB vaccine research [22,24,25].

Given the dual potential of Rv2299c to stimulate DCs and of Ad to induce trained immunity, we hypothesized that a mucosal adenovirus-vectored vaccine expressing Rv2299c (rAd-Rv2299c) could establish a state of trained immunity in DCs, which in turn would orchestrate superior polyfunctional T-cell responses and confer enhanced protection against *Mtb*. To test this, we constructed rAd-Rv2299c and evaluated its capacity to induce DC trained immunity, antigen-specific T-cell responses, and protective efficacy in a murine model of *Mtb* infection. This study aims to advance TB vaccinology by integrating mucosal delivery, vectored antigen presentation, and trained immunity into a single, synergistic vaccine strategy that moves beyond the limitations of current T-cell-centric approaches.

## 2. Materials and Methods

### 2.1. Construction of rAd-Rv2299c

The coding sequence of *Rv2299c* was retrieved from the National Center for Biotechnology Information (NCBI) database, and specific primers were designed using SnapGene 8.0 software. The target gene fragment was amplified by PCR and purified using a PCR Purification Kit (UElandy, Shanghai, China). Subsequently, the fragment was ligated into the linearized pSK17 plasmid via T4 DNA ligase (NEB, Beijing, China). Positive recombinant plasmids were selected and subjected to homologous recombination with the Adsimian-1 adenovirus vector.

### 2.2. Adenovirus Amplification and Purification

The recombinant adenovirus vector was linearized and transfected into HEK 293A cells using PEI as a transfection reagent for viral packaging and amplification. After harvesting, the cells were lysed and the crude viral lysate was purified by cesium chloride (BBI, Shanghai, China) density centrifugation at densities of 1.4 g/L and 1.2 g/L. The resulting viral particles were dialyzed against PBS using a 100 kDa ultrafiltration tube (Merck, Darmstadt, Germany) to remove cesium chloride residues.

### 2.3. Western Blot Analysis

Cells were washed with ice-cold PBS and lysed with RIPA buffer containing phosphatase inhibitor and protease inhibitor cocktail (NCM Biotech, Shanghai, China). Protein concentration was determined by the Bradford method (Beyotime, Shanghai, China). Samples were mixed with loading buffer, separated by 10% SDS-PAGE, and transferred to PVDF membranes. The membranes were blocked with 5% skim milk for 1 h at room temperature and incubated with primary antibodies overnight at 4 °C, and then with horseradish peroxidase (HRP)-conjugated secondary antibodies for 1 h at room temperature. Protein bands were visualized using a Tanon imaging system (Tanon 5200, Shanghai, China).

### 2.4. Mouse Immunization

Female specific-pathogen-free BALB/c mice (5–8 weeks old) were obtained from Huachuang Xinuo Medical Technology Co., Ltd. (Taizhou, China). Under isoflurane anesthesia, mice were immunized via intranasal inhalation with 1 × 10^8^ plaque forming units (PFU) of virus or 50 μL of PBS as a control. Female mice were selected to avoid potential sex-based variability in immune responses.

### 2.5. Mycobacterial Infection and Bacterial Load Assessment

*Mtb* was resuspended to a concentration of 2 × 10^8^ colony forming units (CFU)/mL. Mice were challenged via aerosol exposure with a dose of ~100 CFU/mouse. Four weeks post-infection, lung and spleen were aseptically collected and homogenized. Serial dilutions of homogenates were plated on 7H11 (BD, Franklin Lakes, NJ, USA) solid medium supplemented with OADCs (oleic acid, albumin, dextrose and catalase medium; BD), 0.05% glycerol and a mixture of four antibiotics (40 U/mL polymyxin B, 4 mg/mL amphotericin, 50 mg/mL carbenicillin and 2 mg/mL trimethoprim). Plates were incubated at 37 °C for 3 weeks before enumeration of bacterial colonies.

### 2.6. Histopathological Analysis

Lung tissues were fixed in 4% paraformaldehyde (Absin, Shanghai, China) for 24 h in the dark, followed by paraffin embedding and sectioning. Sections were stained with hematoxylin and eosin and imaged using a TissueFAX200 system (TissueGnostics, Vienna, Austria) The area of inflammatory infiltration was quantified with ImageJ software (ImageJ-win64) and expressed as the percentage of the total imaging field occupied by inflammatory foci.

### 2.7. Preparation of Single-Cell Suspensions from Mouse Lung and Spleen

Lung and spleen tissues were aseptically harvested. To generate single-cell suspensions, lung tissues were minced and digested with collagenase (Absin) and DNase I (Roche, Basal, Switzerland) for 30 min at 37 °C, followed by gentle mechanical dissociation. Meanwhile, spleen tissue was dissociated solely through mechanical disruption using a sterile gauze mesh. The resulting cell suspensions from both tissues were centrifuged at 500× *g* for 5 min. After centrifugation, erythrocytes were lysed with red blood cell lysis buffer (Absin), and the remaining cells were washed, resuspended, and counted for downstream applications.

### 2.8. Induction and In Vitro Training of BMDCs

Bone marrow cells were isolated from the femurs and tibiae of euthanized mice. After flushing the bones using insulin syringes, erythrocytes were lysed, washed, and cultured at 1 × 10^6^ cells/well in 6-well plates with RPMI-1640 medium (Hycolne, UT, USA) containing 10% fetal bovine serum (FBS, Gibco, Grand Island, New York, NY, USA), 10 ng/mL recombinant mouse IL-4 (UA BIOSCIENCE, Nanjing, China), and 20 ng/mL granulocyte-macrophage colony-stimulating factor (GM-CSF, UA BIOSCIENCE). Medium was refreshed every 3 days. On day 7, BMDCs were harvested and seeded into 48-well plates at 1.5 × 10^5^ cells/well. Cells were treated with Ad, rAd-Rv2299c, or PBS for 24 h. After training, cells were washed and cultured for an additional 5 days in fresh RPMI-1640 medium supplemented with 10% FBS and 20 ng/mL GM-CSF, with the medium was refreshed on day 3. Prior to secondary stimulation, cells were harvested, counted and re-seeded at equal densities. They were then incubated overnight (≥16 h) with either lipopolysaccharide (LPS, 50 ng/mL) or heat-killed *Mtb* (HK-*Mtb*, MOI [Multiplicity of Infection] = 10).

### 2.9. Enzyme-Linked Immunosorbent Assay (ELISA)

Uncoated ELISA kits (Thermo Fisher Scientific, Bothell, WA, USA) were used to quantify TNF-α, IL-6, IL-1β, IFN-γ, and IL-17. Briefly, microplates were coated overnight at 4 °C with their respective capture antibodies in coating buffer. After three washes with PBST (PBS containing 0.05% Tween-20), plates were blocked with 1 × blocking buffer for 1 h at room temperature. Following another wash, cellular supernatant or standards (100 μL) were added and incubated for 2 h. Plates were washed five times with PBST and incubated with detection antibodies for 1 h, and then with an HRP-conjugated secondary antibody for 30 min. After a final wash, color development was performed using TMB substrate and stopped with 1 M sulfuric acid. IL-12p70 was measured using a pre-coated ELISA kit (Absin) according to the manufacturer’s protocol. Absorbance was read at 450 nm using a Thermo Scientific microplate reader.

### 2.10. Flow Cytometry

Single-cell suspensions prepared from lung and spleen (1.5 × 10^6^ cells/well) or BMDCS (1.5 × 10^5^ cells/well) were seeded into a U-bottom 96-well plate and stimulated with LPS (50 ng/mL) or HK-*Mtb* (MOI = 10) for 16 h. For intracellular cytokine detection, cells were further incubated for an additional 6 h in the presence of protein transport inhibitors (GolgiStop and GolgiPlug, BD Pharmingen, Franklin Lakes, NJ, USA). Subsequently, cells were washed with PBS and stained with viability dye (BD Pharmingen) for 15 min at room temperature. After washing with staining buffer (PBS containing 2% FBS), cells were incubated with Fc block (BD Pharmingen) for 20 min at 4 °C in the dark. Surface staining was performed using antibody cocktails in 50 μL volume for 20 min at 4 °C in the dark. Following surface staining, cells were fixed and permeabilized using a Fixation/Permeabilization Kit (BD Cytofix/Cytoperm, Franklin Lakes, NJ, USA) for 30 min at 4 °C in the dark. Intracellular cytokines were then stained with corresponding antibodies. Finally, cells were filtered and acquired on an LSRFortessa (BD Biosciences) or CytoFLEX S (Beckman Coulter, Brea, CA, USA) flow cytometer. Data were analyzed using FlowJo software version 10 (FlowJo LLC, Ashland, OR, USA).

### 2.11. Antibodies

The anti-mouse antibodies used in this study were: CD3-eFlour 450 (clone 17A2), MHC-II-APC (clone AF6-120.1) and IL-2-PE (clone JES6-5H4) from eBioscience; CD8-Percp-cy5.5 (clone 53-6.7), IL-17A-AF700 (clone TC11-18H10.1) and IFN-γ-APC (clone XMG1.2) from BioLegend; and CD64-BV650 (clone X54-5/7.1), CD11b-FITC (clone M1/70), CD45-APC-cy7 (clone 30-F11), CD4-FITC (clone RM4-5), CD11c-BV605 (clone HL3), CD86-BV421 (clone GL1), IL-6-PE (clone MP5-20F3), TNF-α-PE-cy7 (clone MP6-XT22), and IL-12-APC (clone P40/P70) from BD Pharmingen.

## 3. Results

### 3.1. Construction and Verification of rAd-Rv2299c

**Figure 1:**
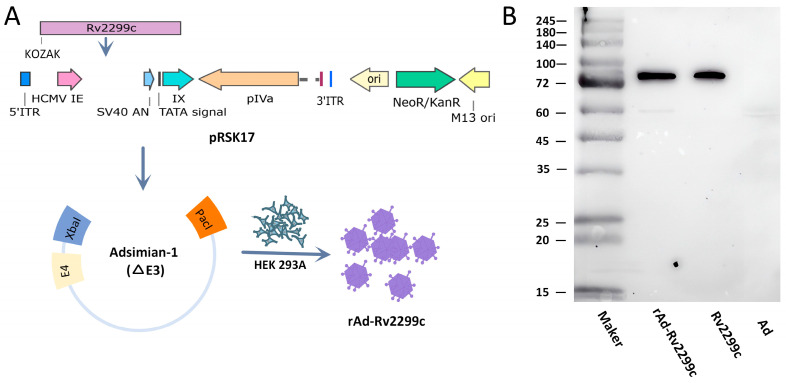
Construction and verification of rAd-Rv2299c. (**A**) Schematic of the rAd-Rv2299c construction process. The *Rv2299c* gene was amplified by PCR from *Mtb* genomic DNA and cloned into the Adsimian-1 vector using the pRSK17 shuttle plasmid. The recombinant plasmid was linearized and transfected into HEK293A cells for packaging and amplification. (**B**) Western blot analysis confirming the expression of the Rv2299c protein by rAd-Rv2299c.

The *Rv2299c* gene from *Mtb* was cloned into the shuttle plasmid pSK17 and subsequently integrated into the adenovirus vector via homologous recombination. The recombinant vector was then transfected into HEK293A cells for viral packaging and amplification (Figure 1A). Sequencing confirmed the integrity of the viral gene sequence (Figure S1A). Western blot analysis showed an Rv2299c protein band with a molecular weight between 72–100 kDa, consistent with the expected size of the purified Rv2299c protein (Figure 1B).

### 3.2. rAd-Rv2299c Induces Trained Immunity in DCs

**Figure 2:**
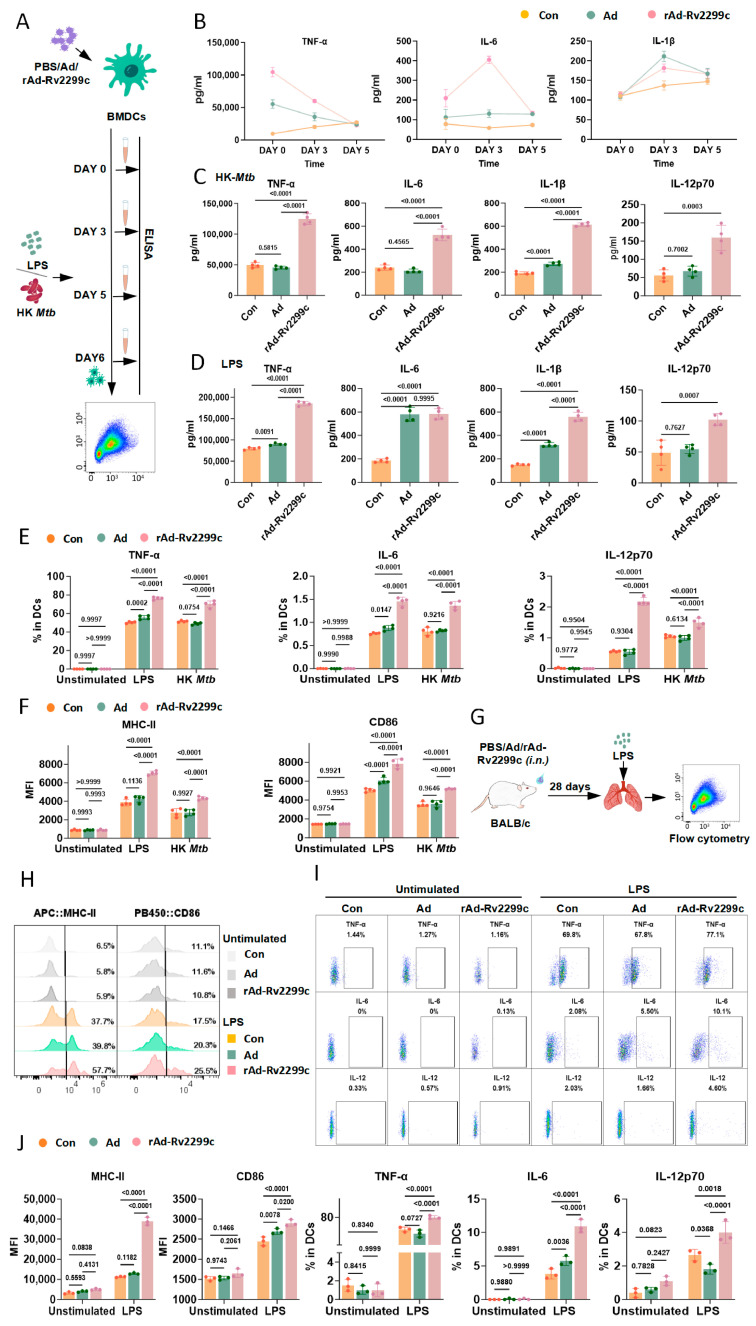
rAd-Rv2299c induces trained immunity in DCs. (**A**) Schematic of in vitro trained immunity model. (**B**) ELISA measurement of TNF-α, IL-6 and IL-1β in BMDCs after stimulation with PBS, Ad, rAd-Rv22299c on day 0, day 3 and day 5 (n = 4). (**C**,**D**) ELISA measurement of TNF-α, IL-6, IL-1β, IL-12p70 in BMDCs after re-stimulation with HK-*Mtb* (**C**) or LPS (**D**) (n = 4). (**E**,**F**) Flow cytometry analysis of BMDCs at baseline (unstimulated) and after HK-*Mtb* or LPS stimulation. Secretion of TNF-α, IL-6, IL-12p70 (**E**) and mean fluorescence intensity (MFI) of MHC-II and CD86 (**F**) were shown (n = 4). (**G**) Schematic of trained immunity evaluation in a murine model. (**H**) Representative histogram of MHC-II and CD86 expression in lung DCs. (**I**) Flow cytometry analysis showing TNF-α, IL-6 and IL-12p70 secretion by lung DCs (n = 3). (**J**) Secretion of TNF-α, IL-6, IL-12p70 in lung DCs at baseline (unstimulated) and after ex vivo LPS stimulation (n = 4). Data are presented as mean ± SEM. *p* values were calculated using two-way ANOVA followed by Dunnett’s multiple comparison test (**B**,**E**,**F**,**J**) or one-way ANOVA followed by Tukey’s post-hoc test (**C**,**D**).

We next assessed the capacity of rAd-Rv2299c to induce trained immunity in DCs using an established in vitro model [26]. BMDCs were trained with PBS, Ad, or rAd-Rv2299c (Figure 2A). After a five-day rest period to allow return to quiescence, as confirmed in Figure 2B,E,F, the cells were restimulated with LPS or HK-*Mtb*. rAd-Rv2299c priming modestly increased the cell quantity of BMDCs (Figure S2A) without affecting apoptosis (Figure S2B) in the trained process; thus, all groups were replated at the same density for secondary stimulation, to preclude the confounding effect of cell quantity. Upon HK-*Mtb* or LPS stimulation, ELISA revealed that rAd-Rv2299c-trained BMDCs produced significantly higher levels of TNF-α, IL-6, IL-1β and IL-12p70 compared to control groups (Figure 2C,D). Ad vector also has a training effect, as evidenced by it significantly elevating TNF-α, IL-6, IL-1β secretion following LPS stimulation, and IL-1β secretion following HK-*Mtb* stimulation (Figure 2C,D). Flow cytometric analysis further demonstrated that rAd-Rv2299c training enhanced the secretion of TNF-α, IL-6, and IL-12p70 (Figure 2E), as well as the surface expression of MHC-II and CD86 (Figure 2F), compared with control groups in response to either LPS or HK-*Mtb* stimulation. These results indicate that rAd-Rv2299c enhances BMDCs’ responsiveness in vitro against heterologous challenge.

We further validated these observations in vivo using a murine model of trained immunity. The mice received intranasal administration of rAd-Rv2299c to target respiratory mucosal immunity [27,28,29,30] (Figure 2G). Upon ex vivo LPS stimulation, lung-derived cells from rAd-Rv2299c-trained mice showed significant upregulation in the expression of MHC-II and CD86, accompanied by elevated secretion of TNF-α, IL-6, and IL-12p70 (Figure 2H–J and Figure S2C,D). Consistent with in vitro observations, training with Ad or rAd-Rv2299c failed to enhance the secretion of TNF-a and CD86/MHC-II expression in lung macrophages, although IL-6 production was increased in rAd-Rv2299c group (Figure S2E,F). These in vivo data confirm that rAd-Rv2299c specifically potentiates DCs’ maturation and functional capacity.

### 3.3. rAd-Rv2299c Immunization Elicits Antigen-Specific Adaptive Responses

**Figure 3:**
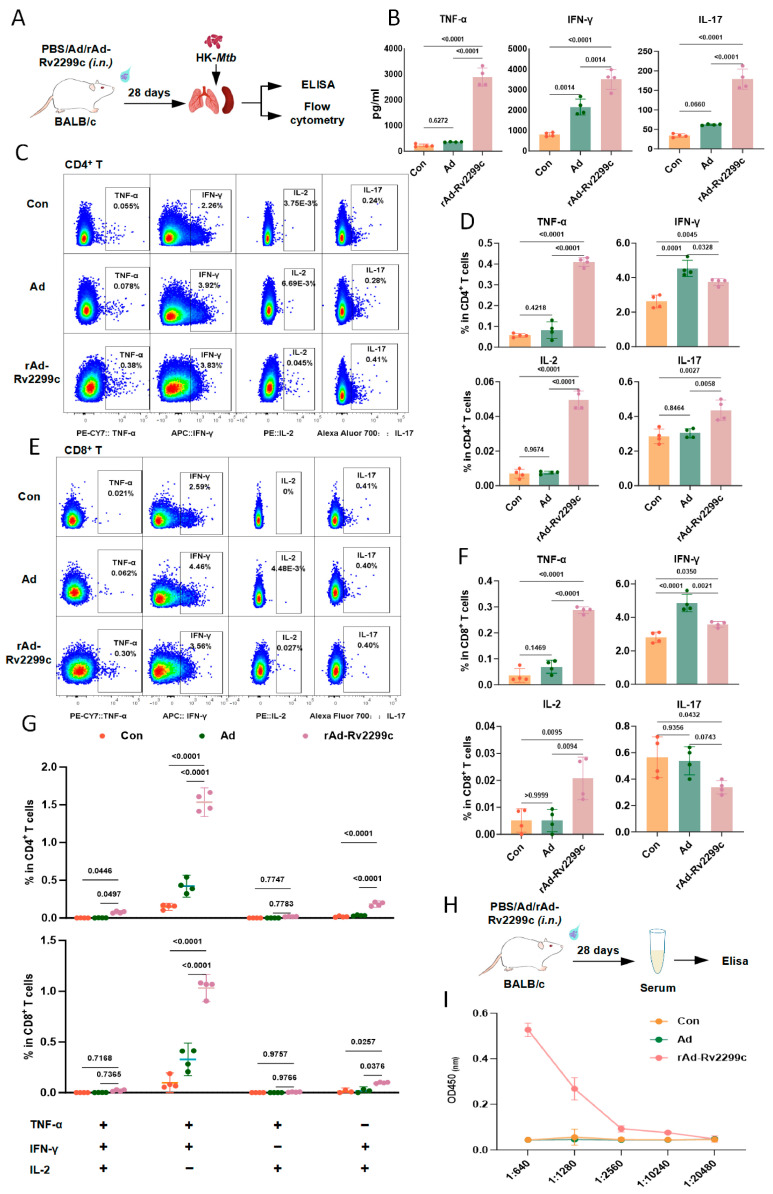
rAd-Rv2299c immunization elicits adaptive immune responses. (**A**) Schematic of immunization and T cell analysis protocol. (**B**) Cytokine levels (TNF-α, IFN-γ, IL-6) in supernatants of HK-*Mtb*-stimulated lung cell cultures. (**C**,**E**) Flow cytometry profiles of TNF-α, IFN-γ, IL-6, and IL-17 production by lung CD4^+^ (**C**) and CD8^+^ T cells (**E**). (**D**,**F**) Quantitative summary of cytokine-positive T cells from (**C**,**E**). (**G**) Statistical analysis of polyfunctional T-cell subsets. (**H**) Schematic of antibody detection experiment. (**I**) Rv2299c-specific serum IgG antibody titers. Data are shown as mean ± SEM and are representative of four independent experiments. *p* values were calculated using one-way ANOVA followed by Tukey’s post-hoc test (**B**,**D**,**F**) or two-way ANOVA followed by Dunnett’s multiple comparison test (**G**,**I**).

We next evaluated adaptive immune responses in immunized mice. Animals received intranasal administration of PBS, Ad, or rAd-Rv2299c, and T-cell immune responses in the spleen and lung were analyzed four weeks later (Figure 3A and Figure S3A). ELISA analysis showed that lung cells from the rAd-Rv2299c training group secreted significantly higher levels of TNF-α, IFN-γ, and IL-17 after HK-*Mtb* stimulation compared with the PBS control group; in contrast, the Ad training showed a significant increase only in IFN-γ secretion (Figure 3B). Flow cytometric analysis further demonstrated that rAd-Rv2299c immunization significantly increased the proportions of Th1-type (TNF-α^+^, IFN-γ^+^, IL-2^+^) and modestly increased Th17 (IL-17^+^) cells among lung CD4^+^ T cells (Figure 3C,D). A similar enhancement was observed in lung CD8^+^ T cells, although no increase in IL-17 secretion was detected (Figure 3E,F). In the spleen, T cell responses largely mirrored those in the lung, with the exception that no significant changes were observed in IL-2 secretion by CD4^+^ T cells or IFN-γ secretion by CD8^+^ T cells (Figure S3B,C). Polyfunctional T cell analysis revealed that rAd-Rv2299c immunization significantly increased the proportions of triple-positive (TNF-α^+^IFN-γ^+^IL-2^+^) and double-positive (TNF-α^+^IFN-γ^+^IL-2^−^, TNF-α^−^IFN-γ^+^IL-2^+^) subsets in the lung CD4^+^ T cells (Figure 3G). Similar increases in these double-positive subsets were also observed among lung CD8^+^ T cells (Figure 3G). Comparable trends were evident in splenic T cells, though the proportion of TNF-α^−^IFN-γ^+^IL-2^+^ CD8^+^ T cells did not change significantly (Figure S3C). Furthermore, Rv2299c-specific antibody titers remained detectable at approximately 1:1000 four weeks post-immunization with rAd-Rv2299c (Figure 3H,I).

Together, these results demonstrate that respiratory mucosal immunization with rAd-Rv2299c effectively elicits antigen-specific T-cell responses, particularly in the lung, and promotes humoral immunity.

### 3.4. rAd-Rv2299c Induces Protection Against Mtb Infection in Mice

**Figure 4:**
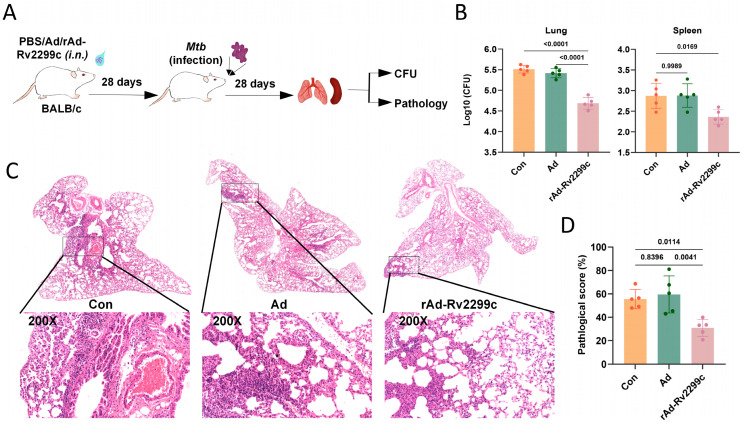
rAd-Rv2299c induces protection against *Mtb* infection in mice. (**A**) Schematic of *Mtb* challenge experiment. (**B**) Bacterial loads in the lung and spleen. (**C**) Representative H&E-stained lung tissue sections. Scale bar = 100 μm. Insets show the original field and its 200× magnified views. (**D**) Quantitative pathology scores, calculated as (area of inflammatory infiltration/total lung area per field) × 100%. Data are shown as mean ± SEM and are representative of five independent experiments. *p* values were calculated by one-way ANOVA followed by Tukey’s post-hoc test (**B**,**D**).

To evaluate the protective efficacy of rAd-Rv2299c, mice were challenged with *Mtb* via aerosol infection four weeks after immunization. Lung and spleen tissues were collected four weeks post-infection for bacterial load assessment (Figure 4A). The rAd-Rv2299c group exhibited significantly reduced bacterial loads in both lung and spleen tissues compared to the control group (Figure 4B). Histopathological analysis further revealed that rAd-Rv2299c immunization markedly attenuated inflammatory cell infiltration in the lung (Figure 4C,D). These findings demonstrate that the rAd-Rv2299c vaccine can elicit effective immune protection against *Mtb* infection in mice.

### 3.5. rAd-Rv2299c Enhances Anti-Mtb Recall Immune Responses

**Figure 5:**
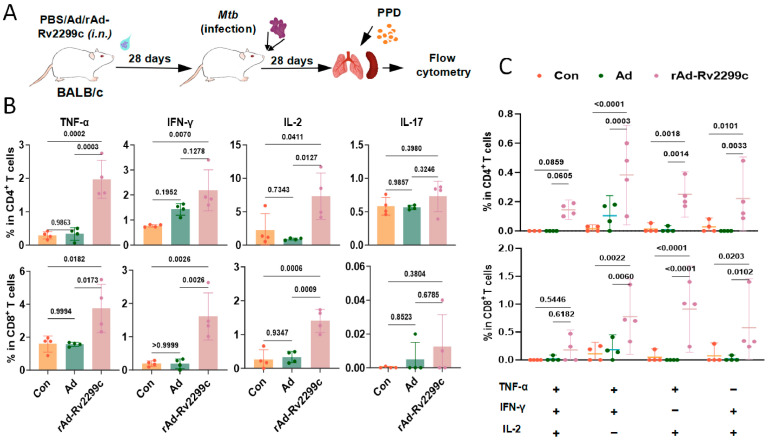
rAd-Rv2299c enhances anti-*Mtb* recall immune responses. (**A**) Schematic of immune recall response detection. (**B**) Frequencies of TNF-α-, IFN-γ-, IL-2-, and IL-17 secretion by lung CD4^+^ and CD8^+^ T cells. (**C**) Statistical analysis of polyfunctional T cell profiles. Data are shown as mean ± SEM and are representative of four independent experiments. *p* values were calculated by one-way ANOVA followed by Tukey’s post-hoc test (**B**) and two-way ANOVA followed by Dunnett’s multiple comparison test (**C**).

To assess memory immune responses, lung single-cell suspensions from immunized mice were restimulated in vitro with purified protein derivative (PPD) of *Mtb* (Figure 5A). Upon re-exposure to PPD, lung CD4^+^ T cells from rAd-Rv2299c-immunized mice exhibited significantly enhanced secretion of TNF-α and IL-2, along with a modest increase in IFN-γ and IL-17 secretion. Similarly, CD8^+^ T cells from the immunized mice exhibited significantly elevated secretion of TNF-α, IFN-γ, and IL-2, with no change in IL-17 production (Figure 5B and Figure S4). Further analysis of polyfunctional T-cell responses showed significant increases in double-positive T-cell subsets (TNF-α^+^IFN-γ^+^IL-2^−^, TNF-α^+^IFN-γ^−^IL-2^+^ and TNF-α^−^IFN-γ^+^IL-2^+^), as well as slight increases in the TNF-α^+^IFN-γ^+^IL-2^+^ subset, within both CD4^+^ and CD8^+^ T cell populations (Figure 5C). These results indicate that rAd-Rv2299c immunization induces a robust and multifunctional memory T-cell response against mycobacterial antigens.

## 4. Discussion

In this study, we demonstrate that intranasal immunization with a chimpanzee adenovirus-vectored vaccine expressing the standalone antigen Rv2299c induces a state of trained immunity in DCs, promotes polyfunctional T-cell responses in the lungs, and confers significant protection against *Mtb* challenge in mice. This mucosal vaccine strategy integrates three key concepts in modern vaccinology: vectored antigen delivery, mucosal barrier immunization, and innate immune training, which offer a novel approach to overcome the limitations of current TB vaccine candidates.

Previous research has shown that fusion proteins containing Rv2299c function as a DC-stimulating adjuvant capable of directly activating DCs; however, these observations were primarily limited to the phenotypic changes induced by acute stimulation [8,9,10]. Our study extends this observation by demonstrating that rAd-Rv2299c effectively induces a “trained immunity” phenotype in DCs. Specifically, after primary immunization and a subsequent resting period, the trained DCs exhibited enhanced expression of MHC-II and CD86, together with increased secretion of TNF-α, IL-6, IL-1β and IL-12p70, upon heterologous challenge. This functional reprogramming appears specific to DCs, as we did not observe a comparable training effect in macrophages. Trained immunity primarily relies on the induction of epigenetic modifications and metabolic reprogramming of innate immune cells [11,31], and various training agents can optimize T-cell-mediated immune responses [32]. Although our study provides phenotypic evidence consistent with trained immunity, mechanistic validation through epigenetic and metabolomic profiling remains a necessary next step to confirm bona fide innate immune memory.

Given previous evidence that Rv2299c fusion protein can activate DCs and enhance T-cell responses in vitro [9,10], we directly evaluated Rv2299c as a standalone protective antigen in a TB vaccine model to assess its capacity to induce antigen-specific T-cell immunity in murine models. We observed that rAd-Rv2299c-immunized mice developed significantly enhanced Th1 and modestly enhanced Th17 responses in lung CD4^+^ T cells upon antigen rechallenge. Notably, we detected a substantial increase in the proportion of polyfunctional T cells, such as TNF-α^+^IFN-γ^+^IL-2^+^ triple-positive populations, which have been widely associated with long-term control of intracellular infections, including TB [33,34,35]. These results suggest that the induction of DC trained immunity was associated with robust and qualitatively superior adaptive immune responses. Importantly, while the empty adenovirus vector alone triggered modest innate activation in vitro, it failed to generate significant antigen-specific T-cell immunity or protection in vivo, underscoring the essential role of Rv2299c as both an immunogen and a trainer of innate immunity.

Protection conferred by rAd-Rv2299c was further validated in a murine challenge model, which showed significant reductions in bacterial loads in the lung and spleen, along with alleviated pulmonary pathology. The protection coincided with potent recall responses, characterized by enhanced cytokine production and polyfunctional profiles in both CD4^+^ and CD8^+^ T cells upon antigen re-exposure. It remains to be determined whether this memory is shaped by DC trained immunity during immunization or infection. Our present study does not provide direct evidence that rAd-Rv2299c-trained DCs are necessary for optimal T-cell immunity. Nevertheless, our data reveal a clear temporal and phenotypic association: in mice immunized with rAd-Rv2299c, we observe both enhanced DC function (upon restimulation) and a pronounced polyfunctional T-cell profile at four weeks post-immunization. Importantly, when these mice are challenged with *Mtb* at the same time point, they exhibit a significant reduction in bacterial burden compared to controls. While these findings do not prove causality, they strongly suggest that the enhanced DC phenotype and the emergence of polyfunctional T cells may function cooperatively to mediate protection.

Our study intentionally focused on evaluating Rv2299c as a standalone antigen in a mucosal delivery platform, and thus did not include a direct comparison with BCG. Nevertheless, contextualizing our findings within the broader TB vaccine landscape is important. BCG, while effective against severe childhood TB, shows limited efficacy in adults and induces primarily systemic rather than lung-resident immunity. In contrast, mucosal rAd-Rv2299c vaccination generated strong local T-cell responses and DC trained immunity in the respiratory tract, highlighting its potential as a complementary or boosting component. Notably, the level of protection achieved by a single mucosal dose of rAd-Rv2299c was comparable to the typical ~1 log CFU reduction observed with BCG in similar murine models in our hand [36,37,38]. Future comparative studies with BCG, especially in prime-boost regimens, will be important to fully contextualize the protective efficacy of rAd-Rv2299c.

While our preclinical data demonstrate promising immunogenicity and protection, several translational challenges must be overcome before clinical advancement can be pursued. First, the safety profile of intranasal adenoviral vectors in humans requires careful evaluation, particularly regarding local inflammatory responses and systemic dissemination. Second, dose optimization (including potential multi-antigen strategies) and vaccination schedules need to be established in non-human primates to identify the minimal effective dose and potential prime-boost strategies. Third, manufacturing scalability and stability of chimpanzee adenovirus vectors must be ensured for large-scale production. Finally, the relevance of our murine findings to human immunity should be validated in human DCs and T-cell assays, and eventually in clinical trials. These steps are essential to translate rAd-Rv2299c from a preclinical candidate to a viable TB vaccine for human use.

The current study has several limitations. First, although our data show functional and phenotypic profiles consistent with trained immunity, we did not provide direct mechanistic evidence. Therefore, the observed “trained” phenotype remains inferred from functional readouts rather than mechanistically proven. Future studies should investigate the underlying epigenetic and metabolic reprogramming in DCs following rAd-Rv2299c exposure to confirm the induction of bona fide trained immunity. Second, given the distinct composition and immune mechanisms of BCG and rAd-Rv2299c, future comparative studies with BCG, especially in prime-boost regimens, will be important to fully contextualize the protective efficacy of rAd-Rv2299c. Third, our study was conducted exclusively in female mice, which may limit the generalizability of findings to both sexes. Future studies should include male mice to assess potential sex differences in vaccine-induced immunity.

## 5. Conclusions

In summary, this study demonstrates that a single intranasal dose of the chimpanzee adenovirus-vectored vaccine rAd-Rv2299c induces a state of trained immunity in DCs and promotes robust, polyfunctional T-cell responses in the lungs, leading to significant protection against aerosol *Mtb* challenge in mice. These findings establish Rv2299c as a promising standalone antigen capable of engaging both innate and adaptive arms of immunity. Thus, our results offer a compelling preclinical foundation for the development of next-generation TB vaccines aimed at achieving durable, lung-resident immunity.

## Data Availability

The data are not publicly available but are available from the corresponding author upon reasonable request.

## Supplementary Materials

The following supporting information can be downloaded at: https://www.mdpi.com/article/10.3390/vaccines14010055/s1, Figure S1: Comparative analysis of sequencing data. Sequence alignment of the Sanger sequencing chromatogram, as visualized in SnapGene, with the reference *Rv2299c* gene sequence from the *Mtb* ATCC genome; Figure S2: Flow cytometry gating strategy and pulmonary macrophage immunophenotype. (A) Quantification of BMDCs following priming with PBS, Ad, or rAd-Rv2299c. (B) Apoptosis of BMDCs on day 5 (unstimulated) and after LPS stimulation. (C) Gating strategy for MHC-II and CD86 expression on lung DCs. (D) Gating strategy for TNF-α, IL-6, and IL-12p70 secretion by lung DCs. (E) Gating strategy for flow cytometric analysis of lung macrophages. (F) MFI of MHC-II and CD86 on lung macrophages, and the frequency of IL-6+ and TNF-α+ macrophages (n = 3). Data are shown as mean ± SEM. *p* values were calculated by one-way ANOVA followed by Tukey’s post hoc test; Figure S3: rAd-Rv2299c immunization establishes T cell memory against *Mtb*. (A) Gating strategy for the flow cytometry analyses presented in Figure 4 and Figure S4. (B,C) Frequency of TNF-α, IFN-γ, IL-6, or IL-17-secreting lung CD4^+^ (B) and CD8^+^ T cells (C). (D) Statistical analysis of polyfunctional T cells profiles. Data are shown as mean ± SEM and are representative of four independent experiments. *p* values were calculated by one-way ANOVA followed by Tukey’s post hoc test (B,C) and two-way ANOVA followed by Dunnett’s multiple comparison test (D); Figure S4: Flow cytometry dot plots. Representative flow cytometry plots of TNF-α, IFN-γ, IL-2, and IL-17 secretion by lung CD4^+^ and CD8^+^ T cells.
