# Supplementary material for: Mucosal Adenovirus-Vectored Rv2299c Vaccine Protects Against Tuberculosis by Inducing Trained Immunity in Dendritic Cells and Polyfunctional T Cells
Source: Vaccines (Basel). 2026 Jan 2;14(1):55. doi: 10.3390/vaccines14010055 (PMC12846401; doi:10.3390/vaccines14010055)
Supplement: Supplementary file 1 [file vaccines-14-00055-s001.zip › vaccines-4022684-supplementary.pdf]

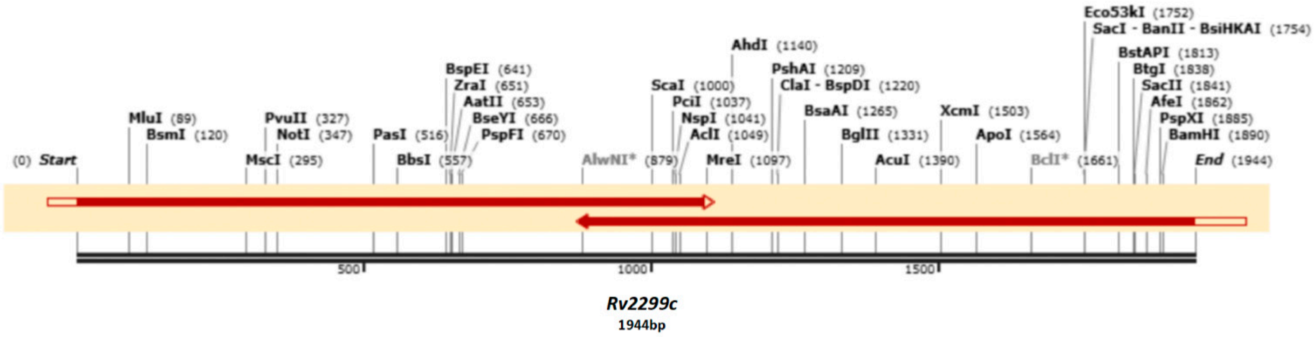

Figure S1. Comparative analysis of sequencing data. Sequence alignment of the Sanger sequencing chromatogram, as visualized in SnapGene, with the reference *Rv2299c* gene sequence from the *Mtb* ATCC genome.

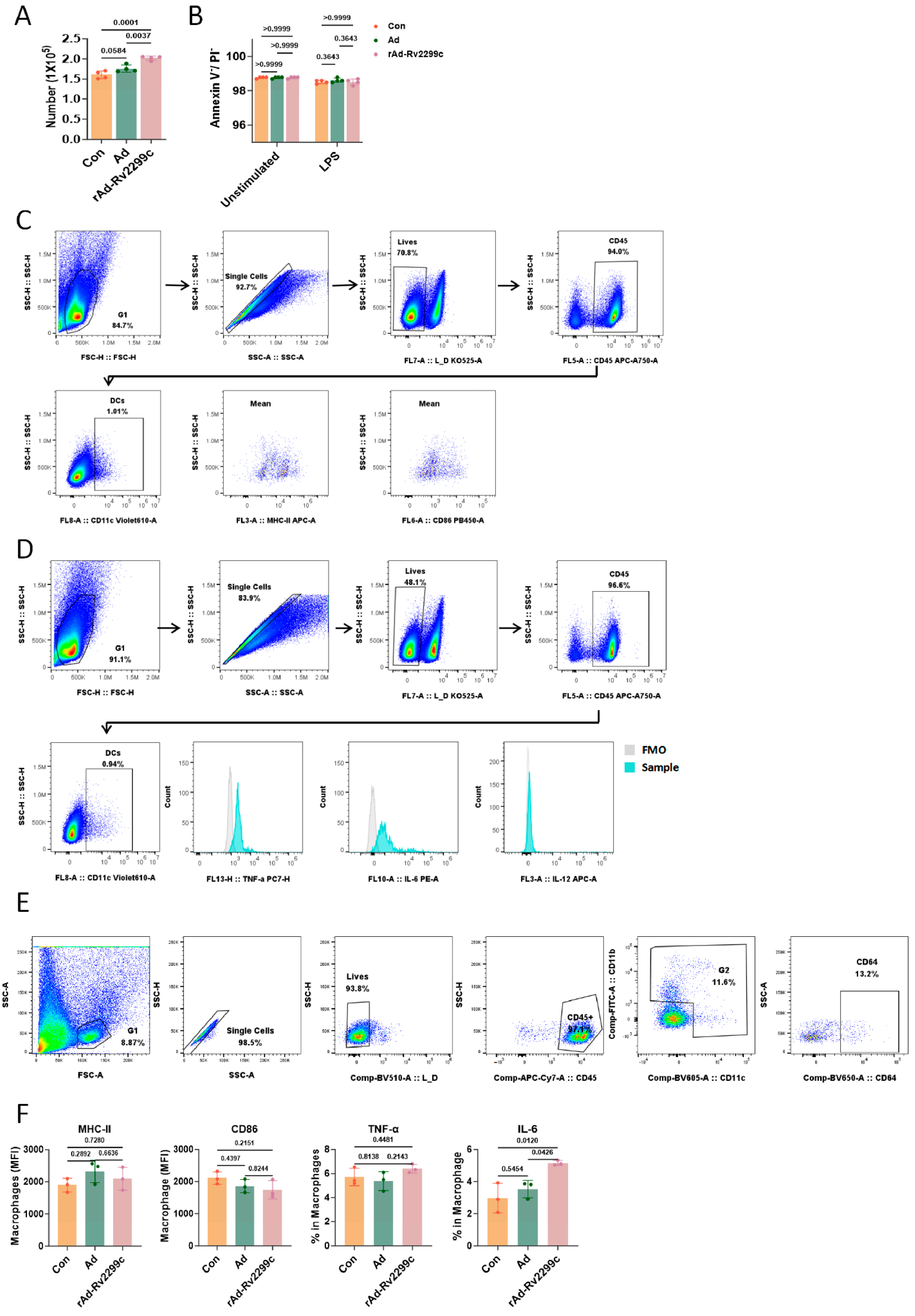

Figure S2. Flow cytometry gating strategy and pulmonary macrophage immunophenotype. (A) Quantification of BMDCs following priming with PBS, Ad, or rAd-Rv2299c. (B) Apoptosis of BMDCs on day 5 (unstimulated) and after LPS stimulation. (C) Gating strategy for MHC-II and CD86 expression on lung DCs. (D) Gating strategy for TNF-α, IL-6, and IL-12p70 secretion by lung DCs. (E) Gating strategy for flow cytometric analysis of lung macrophages. (F) MFI of MHC-II and CD86 on lung macrophages, and the frequency of IL-6$^+$ and TNF-α^+ macrophages (n=3). Data are shown as mean ± SEM. *P values* were calculated by one-way ANOVA followed by Tukey's post hoc test.

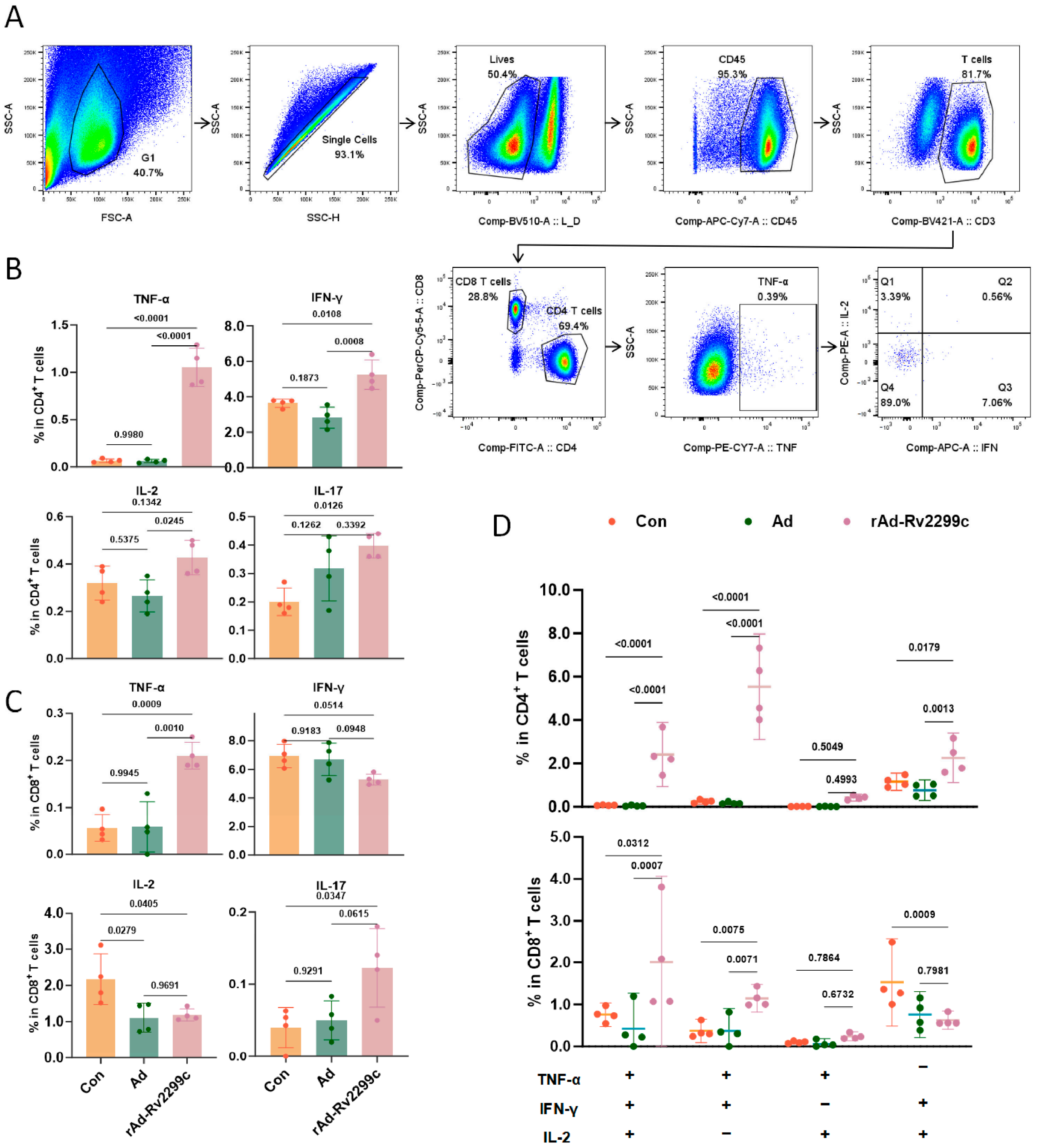

Figure S3. rAd-Rv2299c immunization establishes T cell memory against *Mtb*. (A) Gating strategy for the flow cytometry analyses presented in Fig. 4 and Fig. S4. (B-C) Frequency of TNF-α, IFN-γ, IL-6, or IL-17-secreting lung $CD4^+$(B) and $CD8^+$T cells (C). (D) Statistical analysis of polyfunctional T cells profiles. Data are shown as mean ± SEM and are representative of four independent experiments. *P values* were calculated by one-way ANOVA followed by Tukey's post hoc test (B, C) and two-way ANOVA followed by Dunnett's multiple comparison test (D).

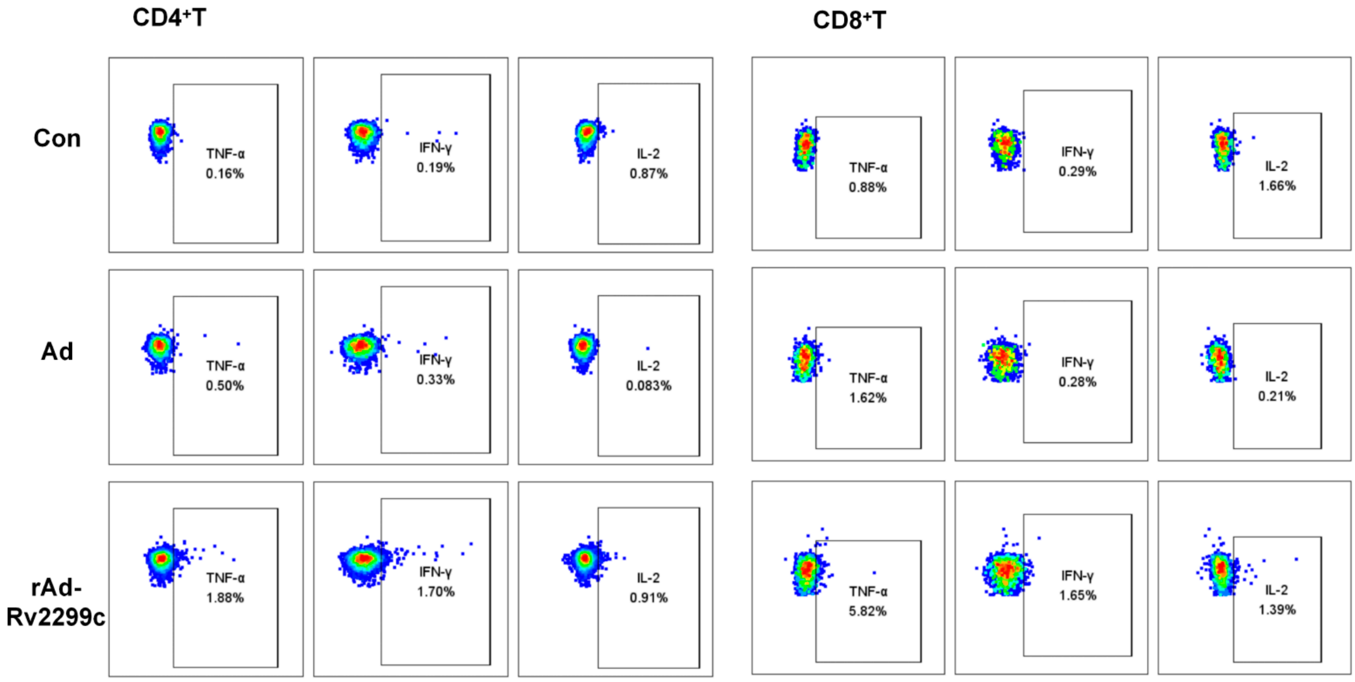

Figure S4. Flow cytometry dot plots. Representative flow cytometry plots of TNF-α, IFN-γ, IL-2, and IL-17 secretion by lung CD4+ and CD8+ T cells.

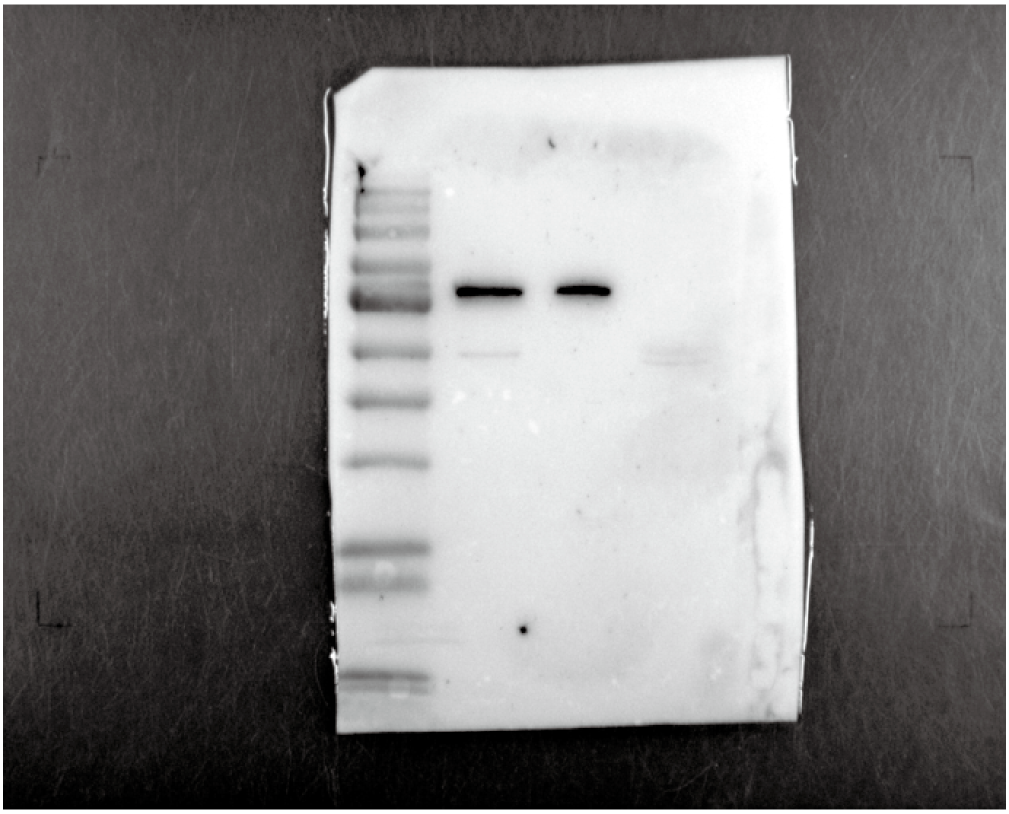

WB original images (Corresponding to Figure 1B).
